# Inter-residue distances derived from fold contact propensities correlate with evolutionary substitution costs

**DOI:** 10.1186/1471-2105-5-153

**Published:** 2004-10-18

**Authors:** Gareth Williams, Patrick Doherty

**Affiliations:** 1 Wolfson Centre for Age-Related Diseases, The Wolfson Wing, Hodgkin Building, Kings College London, London SE1 1UL, UK

## Abstract

**Background:**

The wealth of information on protein structure has led to a variety of statistical analyses of the role played by individual amino acid types in the protein fold. In particular, the contact propensities between the various amino acids can be converted into folding energies that have proved useful in structure prediction. The present study addresses the relationship of protein folding propensities to the evolutionary relationship between residues.

**Results:**

The contact preferences of residue types observed in a representative sample of protein structures are converted into a residue similarity matrix or inter-residue distance matrix. Remarkably, these distances correlate excellently with evolutionary substitution costs. Residue vectors are derived from the distance matrix. The residue vectors give a concrete picture of the grouping of residues into families sharing properties crucial for protein folding.

**Conclusions:**

Inter-residue distances have proved useful in showing the explicit relationship between contact preferences and evolutionary substitution rates. It is proposed that the distance matrix derived from structural analysis may be useful in aligning proteins where remote homologs share structural features. Residue vectors derived from the distance matrix illustrate the spatial arrangement of residues and point to ways in which they can be grouped.

## Background

The large number of protein crystal structures available has naturally led to statistical analyses of protein folding and protein interaction in the hope that these will point to intrinsic residue characteristics and therefore serve as aids in protein fold and interaction prediction. The first such analysis was performed by Miyazawa and Jernigan [1-3], where a statistical protein folding potential, the MJ matrix, was deduced from residue contact propensities in a set of monomeric protein crystal structures. The MJ matrix has been used in various *in silico *folding experiments, reviewed by Jernigan et al [4], and shown to point to the essentially hydrophobic nature of folding interactions [5]. An analysis of the MJ matrix has enabled the reduction in sequence complexity by grouping residues into families [6]. A more detailed study of crystal interactions focusing on hydrogen bond distributions has resulted in mean force potentials that have been successfully used in ligand prediction [7]. It is reasonable therefore to conclude that the statistical approach has pointed to an intrinsic residue:residue potential. In this study we will show that crystal contact statistics can be used to define an inter-residue similarity score that is strongly correlated with an evolutionary substitution cost. As this score is not based on aligning homologous proteins it can serve as a complement to similarity scores derived from substitution matrices when faced with the problem of aligning remotely homologous but structurally similar proteins.

The observation that evolutionarily close residues appear to have similar contact propensities leads us to postulate that the extent of similarity between the contact propensities corresponding to two particular amino acids is related to the ease with which these amino acids can be mutated into each other. We define the contact propensity as 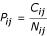, where *N_ij _*is the number of possible pairings between residue type *i *and residue type *j *and *C_ij _*is the number of these parings corresponding to residues in contact. Only non-neighbouring residues on the protein chain are considered and a pair of residues is defined to be in contact if any of their side chain atoms are within a given distance of each other. The difference in contact propensities for two amino acid types can be measured by their rms difference and we define 
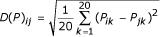
 as our amino acid difference measure or distance matrix.

If we have really got a measure of the distances between residue types then it should follow that residues sharing physical properties are close together. More crucially, we expect that residues that are distant according to *D(P) *will be difficult to mutate into one another and vice versa. This is because the factors involved in determining mutation rates are dominated by those affecting the structural integrity of the protein. Such factors are residue hydropathy, size, charge and etc. Substitution matrices such as PAM and Blosum are determined from mutation rates in aligned protein sequences [8,9]. We can define an amino acid distance matrix in a similar way to *D_ij _*above.

That is, 
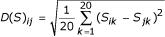
, where *S *is the substitution rate matrix. We show below that *D*(*P*) is indeed strongly correlated with *D*(*S*). It must be stressed that *D*(*P*) and *D*(*S*) are independently derived, with one based on structure and the other on sequence alignment. Their strong correlation is indicative of the validity of our definition of inter-residue distance.

Relating amino acids through a structurally defined distance measure should provide a useful tool for aligning remotely homologous protein sequences. Also, a distance measure naturally leads us to look for a vector representation of the amino acids. In much the same way as average hydropathy plots are useful in structural analysis we expect that average vector profiles will also pick out various structural features. Given a vector for each residue type we can visualise the residues in some abstract space and look for natural groupings of residues and thereby find ways of reducing the effective number of residues.

## Results

A representative set of crystal structures was compiled from the PDBselect25 database [10], which contains structures sharing at most 25% sequence homology. We made sure that side chain coordinates were defined and restricted chain lengths to be greater than 50 and less than 500 residues long. In short we arrived at 1073 structures and performed the statistical analysis on these. Residues are held to be in contact if any of their respective side chain atoms are within a given distance of each other. Only residue pairs that are not neighbours along the chain are considered in the analysis of intra-molecular contacts.

**Figure 1:**
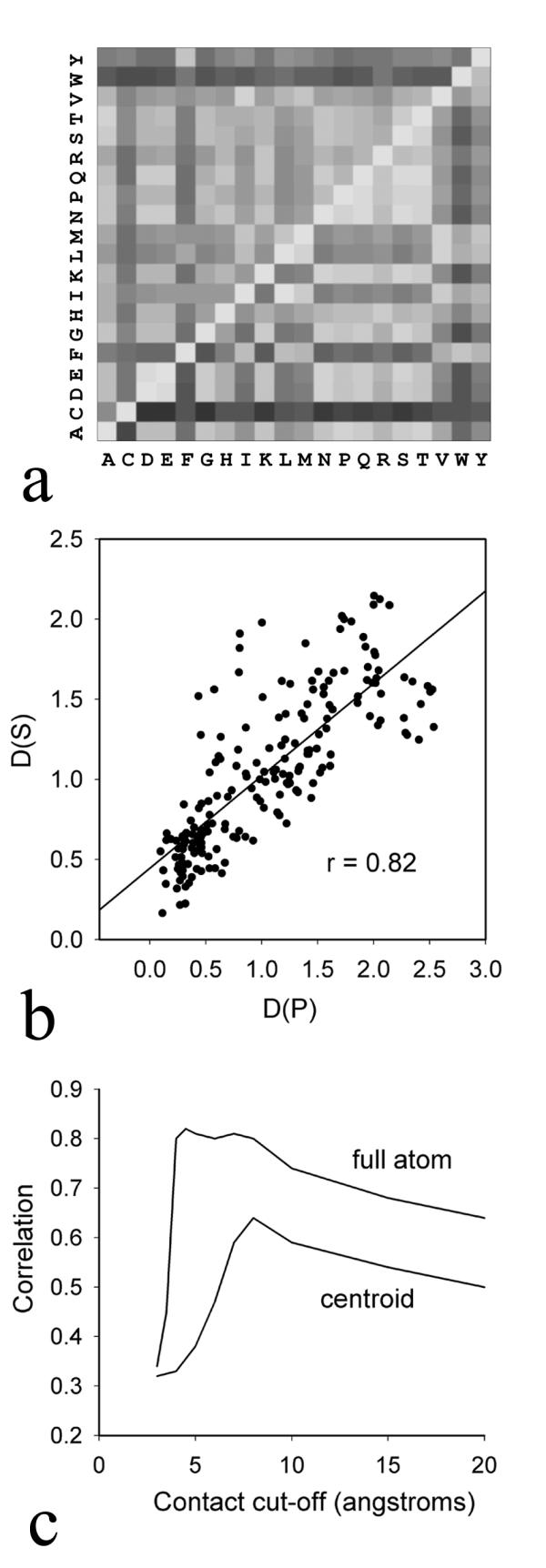
Comparison of the distance matrices derived from intra-molecular crystal contacts and from the PAM250 evolutionary substitution rates. In (a) *D*(*S*) is plotted in the upper triangle and *D*(*P*) in the lower, larger distances correspond to lighter shades. We have scaled *D *to average unity i.e. <*D*> = 1. It is apparent that the two matrices have a similar pattern. The correlation is shown explicitly in (b). The extent of correlation varies with the contact cut-off distance and peaks at a cut-off of 4.5Å, see (c). When contacts are scored by residue centroid proximity then the correlation is less strong, see (c).

**Figure 2:**
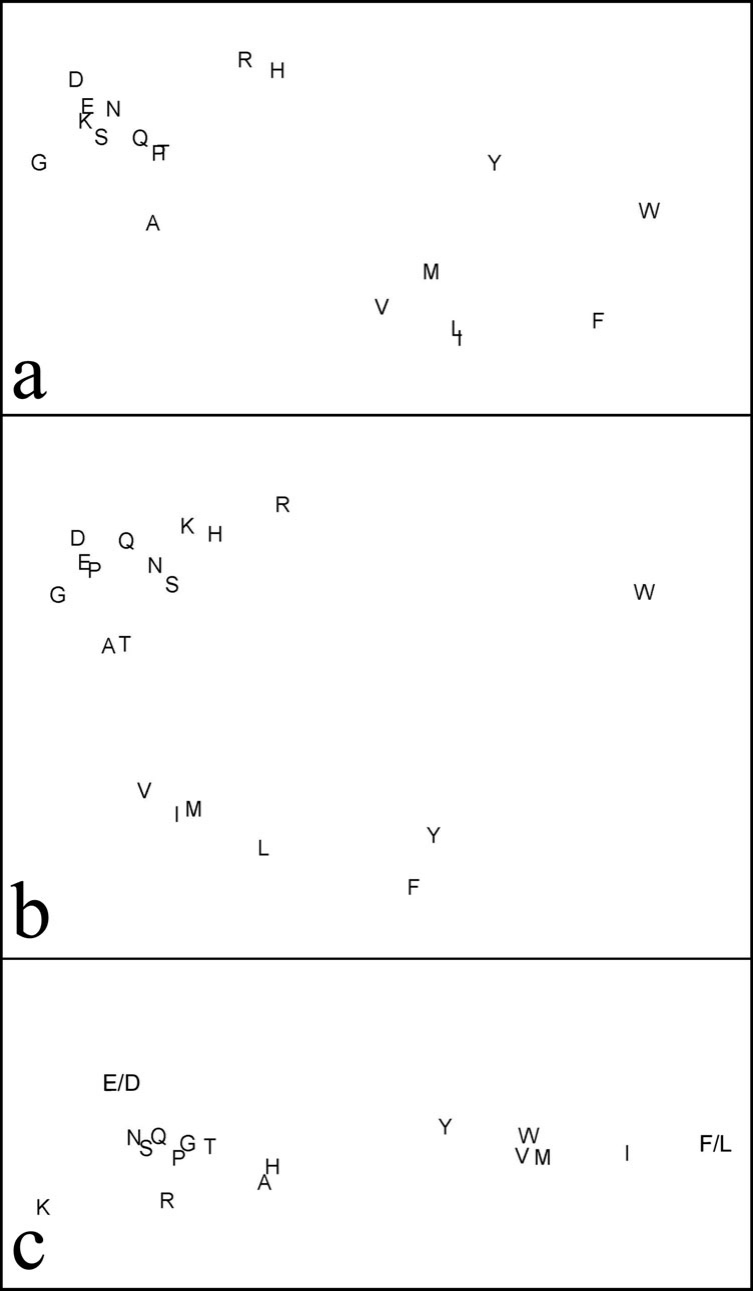
A two-dimensional representation of the amino acid vectors. The contact propensity distance matrix derived vectors are shown in (a) and it is clear that residues with similar hydropathies and sizes are grouped together. A similar vector space can be obtained from an evolutionary substitution rate matrix and this is shown in (b). The residue positions are similar in (a) and (b), but in (a) residues with opposite hydropathies appear to lie further apart. In contrast, the vector space derived from the MJ energy matrix appears to be roughly linear. Here, the residues group in essentially the same way as reported by Wang & Wang [6].

As explained above the contact propensities can be converted to a distance matrix *D*(*P*). If this matrix is really a measure of residue similarity then we should be able to correlate it with an equivalent matrix constructed from an evolutionary substitution rate matrix. In what follows we will take PAM250 as the substitution matrix. In Figure 1a we show the contour plots of *D*(*S*) in the top triangle and *D*(*P*) in the bottom triangle for a contact cut-off of 4.5Å, where the pearson correlation (r) is maximal, with *r *= 0.82. See additional table 1 for explicit values of *P *and *D*(*S*). The correlation can be seen explicitly in Figure 2b. The extent of correlation is roughly constant over a large range of cut-offs (4~8Å) and only drops when the cut-off is small and contacts are rare or when the cut-off is so big that non-interacting residues are scored, see Figure 1c. We expect that, due to the wide range of side chain sizes, a full atom representation is more accurate than a centroid representation and we find that the centroid *D*(*P*) is consistently less well correlated with *D*(*S*), peaking at *r *= 0.64 for a cutoff of 8Å, see Figure 1c.

We have defined inter-residue distances and this implies that there must be a vector representation for the residues. In this case the distance matrix will be 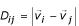, where 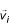 are the residue vectors. Explicitly, the vectors are defined such that 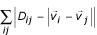 is minimal. When these vectors are derived it becomes clear that Cysteine is quite separate from the other residues in this property space and this maybe due to the unique role played by Cysteine in stabilising folds. Though it must be made clear that the distance matrix is independent of the frequency of an amino acid contacting its own kind and therefore does not count Cysteine bridges in the structures. Without Cysteine the distance matrix can be projected onto a plane i.e. the vectors can be taken to be two-dimensional and this vector space is illustrated in Figure 2a. It is a reasonable postulate that neighbouring residues share physical characteristics and we see similar residue groupings in a standard amino acid Venn diagram [11]. Indeed the vector grouping may serve as a way of reducing the effective amino acid number [6]. It is illuminating to compare vector spaces derived from other statistical analyses. The substitution rate vector space Figure 2b is, as expected, similar to that of the contact propensity vector space, though in *D*(*P*) residues with opposite hydropathies tend to be further apart. This is consistent with hydropathy playing a pivotal role in protein folding. In contrast, the MJ energy matrix vector space is shown in Figure 2c and here the residues effectively lie on a line, which is in accordance with Li et al [5], where the MJ matrix was shown to be dominated by its principal eigenvector reduction. However, for the contact propensity and evolutionary substitution rate spaces, a lot of information is lost in such a linear projection and our analysis clearly points to a higher dimensional representation of the residues. Nonetheless, to make a concrete comparison of our vectors with existing scalar representations of amino acid properties we are forced to project our vectors onto a line. See additional table 2 for the explicit vector components of the contact propensity, substitution rate and MJ energy matrices.

The dominant driving force of folding, at least in defining the crude fold, is hydrophobicity and it is apparent that residues with similar hydrophobicities are grouped together. It also seems that residues of similar size tend to be close in this space. To make a direct comparison between existing residue scales and our vectors we can project the residue vectors onto a line. Here the amino acid scalars, one-dimensional vectors, *d*_*i *_are defined such that 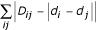 is minimal. We find that these distance matrix derived scalars have a correlation of 0.65 with the Kyte-Doolittle hydrophobicity scale [12] and a correlation of 0.53 with an amino acid volume scale [13]. It is clear therefore that the residue vectors capture a combination of factors determining protein folding.

It is worth noting that a scalar reduction of the distance matrix can be got by a principal eigenvector analysis. In a principal eigenvector reduction of the contact propensity matrix we have *P*_*ij *_= *λe*_*i*_*e*_*j*_, where *λ *is the principal eigenvalue and *e_i _*is the principal eigenvector, and consequently our distance matrix has a scalar representation, 
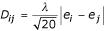
. It is not surprising that the eigenvector is closely related to our scalar, in fact *r*(*e*,*d*) = 0.98. There are many hydrophobicity scales in the literature [14] and some are remarkably similar to our scalar amino acid representation, for example *r *= 0.95 for Wertz & Scheraga scale [15]. However, the highly correlated scales are derived from residue burial statistics in protein structures and are therefore not independent of our statistic.

## Discussion

We have generated full atom residue:residue contact propensity profiles for intra-molecular interactions from a non-redundant crystal structure database. Recasting the contact propensity matrix as a distance matrix we see that close residues are those with a low evolutionary substitution cost. The structure derived distance measures can serve as additional scores when aligning proteins where remote homologs share structural features. The distance matrix led us naturally to derive effective residue vectors. We found that residues sharing similar physical characteristics, such as hydrophobicity and volume, are grouped together. In contrast to the MJ matrix analysis, we find that a scalar representation for the residues is inadequate to capture the complexity of the propensity distance matrix. The most successful scalar representation for the amino acid residues has been the hydropathy scale. Representing a sequence as a smoothed hydropathy profile through wavelet analysis or simple averaging has resulted in many effective analytical tools, such as periodic structure prediction [16], remote homology analysis, helix prediction [14], transmembrane prediction [17] etc. It is then probable that a higher dimensional vector representation of the amino acids may lead to a more subtle sequence analysis. The distance matrix may also serve as an additional tool in sequence alignment as it gives one a measure of the structural cost of residue mutation and this is an idea we hope to pursue in a future study.

## Conclusions

In this study we have shown that inter-residue distance matrices and residue vectors allow us to make an explicit connection between amino acid interaction preferences observed in protein structures and amino acid evolutionary substitution costs. When problems are encountered with aligning structurally related proteins that are remote homologs then the structurally defined distance matrix may prove to be an effective supplement to existing substitution rate derived matrices. The distance matrix leads naturally to an amino acid vector representation. Projecting the vectors onto a two-dimensional plane illustrates ways in which the amino acids can be grouped and their effective number thereby reduced.

## Methods

The database used in the present study was compiled from the PDBselect25 [10] list of representative proteins with known crystal structure that share less than 25% sequence homology. The structural coordinates were downloaded by automated ftp from the NCBI protein data bank. All programmes were written in C, compiled with Metrowerks CodeWarrior and run on a PC. In brief, the contact propensity statistics were compiled by reading the amino acid sequence and atomic coordinates for the specified chain of each pdb structure file in turn. The number of possible pairings of amino acid type *i *with amino acid type *j*, *N_ij _*were counted together with the number of these pairings corresponding to a pair with side chain atoms within a given distance of each other, *C_ij_*. The contact propensity matrix is given by 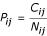. The residue vectors were defined such that 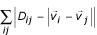 is minimal. The minimisation was carried out by a standard Newton-Raphson steepest descent iteration.

## Supplementary Material

Additional table 1 Contact propensities and distance matrix derived from the structural database with contact cut-off set at 4.5Å. Click here for file

Additional table 2 Two dimensional residue vector components derived from the contact propensity distance matrix, the PAM250 distance matrix and the MJ distance matrix. Click here for file

## References

[B1] Miyazawa S, Jernigan RL (1985). Estimation of effective interresidue contact energies from protein crystal structures: quasi-chemical approximation. Macromolecules.

[B2] Miyazawa S, Jernigan RL (1996). Residue-residue potentials with a favorable contact pair term and an unfavorable high packing density term, for simulation and threading.. J Mol Biol.

[B3] Miyazawa S, Jernigan RL (1999). An empirical energy potential with a reference state for protein fold and sequence recognition.. Proteins.

[B4] Jernigan RL, Bahar I (1996). Structure-derived potentials and protein simulations.. Curr Opin Struct Biol.

[B5] Li H, Tang C, Wingreen NS (1997). Nature of Driving Force for Protein Folding: A Result From Analyzing the Statistical Potential. Phys Rev Lett.

[B6] Wang J, Wang W (2002). Grouping of residues based on their contact interactions.. Phys Rev E Stat Nonlin Soft Matter Phys.

[B7] Grzybowski BA, Ishchenko AV, Kim CY, Topalov G, Chapman R, Christianson DW, Whitesides GM, Shakhnovich EI (2002). Combinatorial computational method gives new picomolar ligands for a known enzyme.. Proc Natl Acad Sci.

[B8] Dayhoff MO, Schwartz RM, Orcutt BC, Dayhoff MO (1978). A model of evolutionary change in proteins.. In Atlas of Protein Sequence and Structure.

[B9] Henikoff S, Henikoff JG (1992). Amino Acid Substitution Matrices from Protein Blocks.. Proc Natl Acad Sci.

[B10] Hobohm U, Sander C (1994). Enlarged representative set of protein structures.. Protein Sci.

[B11] Zvelebil MJ, Barton GJ, Taylor WR, Sternberg MJ (1987). Prediction of protein secondary structure and active sites using the alignment of homologous sequences.. J Mol Biol.

[B12] Kyte J, Doolittle RF (1982). A simple method for displaying the hydropathic character of a protein.. J Mol Biol.

[B13] Zamyatnin AA (1972). Protein volume in solution.. Prog Biopsy Mol Boil.

[B14] Cornette JL, Cease KB, Margalit H, Spouge JL, Berzofsky JA, DeLisi C (1987). Hydrophobicity scales and computational techniques for detecting amphipathic structures in proteins.. J Mol Biol.

[B15] Wertz DH, Scheraga HA (1978). Influence of water on protein structure. An analysis of the preferences of amino acid residues for the inside or outside and for specific conformations in a protein molecule.. Macromolecules.

[B16] Murray KB, Gorse D, Thornton JM (2002). Wavelet transforms for the characterization and detection of repeating motifs.. J Mol Biol.

[B17] Kim J, Moriyama EN, Warr CG, Clyne PJ, Carlson JR (2000). Identification of novel multi-transmembrane proteins from genomic databases using quasi-periodic structural properties.. Bioinformatics.

