# Supplementary material for: Inter-residue distances derived from fold contact propensities correlate with evolutionary substitution costs
Source: BMC Bioinformatics. 2004 Oct 18;5:153. doi: 10.1186/1471-2105-5-153 (PMC526251; doi:10.1186/1471-2105-5-153)
Supplement: Additional table 1 — Contact propensities and distance matrix derived from the structural database with contact cut-off set at 4.5Å. [file 1471-2105-5-153-S1.pdf]

	A	C	D	E	F	G	H	I	K	L	M	N	P	Q	R	S	T	V	W	Y
A	**0.50**	2.31	0.59	0.52	1.61	0.46	0.68	1.16	0.45	1.13	0.99	0.42	0.29	0.30	0.60	0.35	0.27	0.86	1.74	1.22
C	**0.79**	**8.20**	2.53	2.51	2.02	2.54	2.06	2.07	2.48	2.03	1.99	2.42	2.27	2.35	2.28	2.41	2.29	2.04	2.00	1.97
D	**0.28**	**0.45**	**0.29**	0.11	2.01	0.40	0.80	1.61	0.45	1.59	1.41	0.29	0.48	0.37	0.73	0.33	0.46	1.33	2.06	1.51
E	**0.29**	**0.40**	**0.24**	**0.25**	1.95	0.38	0.78	1.54	0.44	1.51	1.34	0.27	0.42	0.32	0.70	0.29	0.41	1.25	2.00	1.45
F	**1.28**	**2.16**	**0.51**	**0.61**	**3.67**	2.04	1.40	0.54	1.94	0.52	0.63	1.86	1.64	1.73	1.55	1.85	1.63	0.79	0.43	0.67
G	**0.26**	**0.45**	**0.32**	**0.23**	**0.65**	**0.19**	0.91	1.61	0.31	1.58	1.42	0.32	0.46	0.37	0.78	0.25	0.46	1.31	2.14	1.60
H	**0.57**	**1.48**	**1.06**	**0.89**	**1.38**	**0.41**	**1.93**	1.10	0.75	1.06	0.86	0.65	0.53	0.58	0.38	0.67	0.52	0.86	1.39	0.85
I	**1.13**	**1.56**	**0.39**	**0.53**	**2.78**	**0.43**	**0.97**	**2.83**	1.51	0.09	0.34	1.46	1.25	1.33	1.20	1.44	1.22	0.31	0.81	0.62
K	**0.31**	**0.52**	**0.86**	**0.91**	**0.70**	**0.26**	**0.53**	**0.56**	**0.28**	1.49	1.32	0.26	0.39	0.30	0.53	0.23	0.38	1.22	2.00	1.46
L	**1.07**	**1.65**	**0.38**	**0.48**	**2.86**	**0.43**	**0.99**	**2.64**	**0.56**	**2.59**	0.27	1.43	1.21	1.30	1.18	1.41	1.19	0.29	0.80	0.59
M	**0.98**	**1.59**	**0.41**	**0.52**	**2.73**	**0.57**	**1.12**	**2.12**	**0.57**	**2.10**	**2.06**	1.24	1.02	1.12	0.98	1.24	1.02	0.28	0.80	0.46
N	**0.36**	**0.61**	**0.58**	**0.48**	**0.78**	**0.39**	**0.82**	**0.51**	**0.54**	**0.56**	**0.67**	**0.73**	0.29	0.14	0.52	0.12	0.25	1.16	1.91	1.36
P	**0.41**	**0.90**	**0.37**	**0.44**	**1.07**	**0.33**	**0.79**	**0.76**	**0.36**	**0.72**	**0.84**	**0.56**	**0.53**	0.19	0.46	0.28	0.23	0.96	1.72	1.18
Q	**0.40**	**0.73**	**0.48**	**0.45**	**0.91**	**0.37**	**0.75**	**0.71**	**0.55**	**0.72**	**0.84**	**0.61**	**0.56**	**0.63**	0.47	0.15	0.14	1.03	1.80	1.25
R	**0.50**	**0.87**	**1.37**	**1.34**	**1.22**	**0.50**	**1.02**	**0.93**	**0.48**	**0.92**	**0.89**	**0.85**	**0.79**	**0.81**	**0.86**	0.56	0.44	0.95	1.55	1.01
S	**0.36**	**0.66**	**0.48**	**0.45**	**0.86**	**0.31**	**0.80**	**0.59**	**0.41**	**0.60**	**0.62**	**0.54**	**0.43**	**0.56**	**0.71**	**0.49**	0.24	1.14	1.93	1.38
T	**0.51**	**0.84**	**0.47**	**0.53**	**1.12**	**0.37**	**0.89**	**0.94**	**0.54**	**0.88**	**0.88**	**0.68**	**0.57**	**0.67**	**0.84**	**0.57**	**0.73**	0.93	1.71	1.16
V	**0.99**	**1.46**	**0.37**	**0.45**	**2.48**	**0.40**	**0.91**	**2.29**	**0.48**	**2.17**	**1.71**	**0.49**	**0.68**	**0.65**	**0.78**	**0.56**	**0.85**	**2.08**	1.00	0.64
W	**1.29**	**2.38**	**0.92**	**1.06**	**3.44**	**0.87**	**1.86**	**2.58**	**1.02**	**2.68**	**2.72**	**1.13**	**1.66**	**1.31**	**1.75**	**1.12**	**1.28**	**2.24**	**3.48**	0.58
Y	**1.10**	**1.83**	**1.00**	**1.03**	**2.54**	**0.72**	**1.64**	**2.06**	**1.05**	**2.01**	**2.06**	**0.99**	**1.47**	**1.11**	**1.58**	**0.87**	**1.01**	**1.73**	**2.89**	**2.12**

Additional table 1: In the bottom half and diagonal, in bold, we have the contact propensities P_{ij}, normalised to average unity, derived from a database of 1073 non-redundant protein structures with contact cut-off set at 4.5Å. The top half shows the corresponding inter-residue distances $D_{ij} = \sqrt{\frac{1}{20}\sum_k \left(P_{ik} - P_{jk}\right)^2}$, again normalised to unity. The distance matrix is zero along the diagonal.
