# Supplementary material for: Inter-residue distances derived from fold contact propensities correlate with evolutionary substitution costs
Source: BMC Bioinformatics. 2004 Oct 18;5:153. doi: 10.1186/1471-2105-5-153 (PMC526251; doi:10.1186/1471-2105-5-153)
Supplement: Additional table 2 — Two dimensional residue vector components derived from the contact propensity distance matrix, the PAM250 distance matrix and the MJ distance matrix. [file 1471-2105-5-153-S2.pdf]

	Contact Propensity r = 0.9995		PAM250 r = 0.9867		MJ r = 0.99995	
	X	Y	X	Y	X	Y
A	0.47	0.16	-0.33	-0.23	0.05	0.25
D	0.77	-0.42	-0.20	-0.67	0.76	0.45
E	0.72	-0.31	-0.23	-0.57	0.76	0.44
F	-1.31	0.58	0.20	1.24	-1.05	-1.16
G	0.93	-0.09	-0.39	-0.51	0.37	0.38
H	-0.04	-0.45	0.27	-0.39	0.07	0.19
I	-0.77	0.64	-0.43	0.50	-0.87	-0.91
K	0.73	-0.25	0.19	-0.47	0.58	0.98
L	-0.75	0.60	-0.22	0.79	-1.06	-1.16
M	-0.64	0.37	-0.39	0.50	-0.64	-0.63
N	0.62	-0.30	0.00	-0.41	0.54	0.52
P	0.44	-0.12	-0.21	-0.52	0.35	0.44
Q	0.52	-0.18	-0.05	-0.55	0.48	0.45
R	0.09	-0.49	0.56	-0.35	0.26	0.59
S	0.67	-0.19	0.02	-0.31	0.47	0.51
T	0.42	-0.12	-0.26	-0.20	0.30	0.32
V	-0.45	0.51	-0.51	0.34	-0.58	-0.58
W	-1.51	0.14	1.60	0.71	-0.53	-0.64
Y	-0.91	-0.06	0.38	1.10	-0.28	-0.43

Additional table 2: The components of the two-dimensional residue vectors. For the given statistic we generate an inter-residue distance matrix D_{ij} and the vectors $\vec{v}_i$ are then defined such that they minimise $\left|D_{ij}(P) - \left|\vec{v}_i - \vec{v}_j\right|\right|$. The distance matrix is invariant under translations and rotations of the vectors and we have taken the vector sum to be at the origin, $\sum_i \vec{v}_i = \vec{0}$. The correlation factors are between the distance matrix and its vector representation $r\left(D_{ij}, \left|\vec{v}_i - \vec{v}_j\right|\right)$.
